# A designed ankyrin-repeat protein that targets Parkinson’s disease-associated LRRK2

**DOI:** 10.1016/j.jbc.2024.107469

**Published:** 2024-06-12

**Authors:** Verena Dederer, Marta Sanz Murillo, Eva P. Karasmanis, Kathryn S. Hatch, Deep Chatterjee, Franziska Preuss, Kamal R. Abdul Azeez, Landon Vu Nguyen, Christian Galicia, Birgit Dreier, Andreas Plückthun, Wim Versees, Sebastian Mathea, Andres E. Leschziner, Samara L. Reck-Peterson, Stefan Knapp

**Affiliations:** 1 Institute of Pharmaceutical Chemistry, Goethe-Universität, Frankfurt, Germany; 2 Structural Genomics Consortium (SGC), Buchmann Institute for Life Sciences, Goethe-Universität, Frankfurt, Germany; 3 Aligning Science Across Parkinson’s (ASAP), Chevy Chase, Maryland, USA; 4 Department of Cellular and Molecular Medicine, School of Medicine, University of California, San Diego, La Jolla, California, USA; 5 Department of Molecular Biology, School of Biological Sciences, University of California, San Diego, La Jolla, California, USA; 6 VIB-VUB Center for Structural Biology, Brussels, Belgium; 7 Structural Biology Brussels, Vrije Universiteit Brussel, Brussels, Belgium; 8 Department of Biochemistry, University of Zurich, Zurich, Switzerland; 9 Department of Cell and Developmental Biology, School of Biological Sciences, University of California, San Diego, La Jolla, California, USA; 10 Howard Hughes Medical Institute, Chevy Chase, Maryland, USA

**Keywords:** Parkinson’s disease, LRRK2, DARPin, WD40, microtubule, kinase, cryo-electron microscopy, kinase inhibitor, Rab8a

## Abstract

Leucine rich repeat kinase 2 (LRRK2) is a large multidomain protein containing two catalytic domains, a kinase and a GTPase, as well as protein interactions domains, including a WD40 domain. The association of increased LRRK2 kinase activity with both the familial and sporadic forms of Parkinson’s disease has led to an intense interest in determining its cellular function. However, small molecule probes that can bind to LRRK2 and report on or affect its cellular activity are needed. Here, we report the identification and characterization of the first high-affinity LRRK2-binding designed ankyrin-repeat protein (DARPin), named E11. Using cryo-EM, we show that DARPin E11 binds to the LRRK2 WD40 domain. LRRK2 bound to DARPin E11 showed improved behavior on cryo-EM grids, resulting in higher resolution LRRK2 structures. DARPin E11 did not affect the catalytic activity of a truncated form of LRRK2 *in vitro* but decreased the phosphorylation of Rab8A, a LRRK2 substrate, in cells. We also found that DARPin E11 disrupts the formation of microtubule-associated LRRK2 filaments in cells, which are known to require WD40-based dimerization. Thus, DARPin E11 is a new tool to explore the function and dysfunction of LRRK2 and guide the development of LRRK2 kinase inhibitors that target the WD40 domain instead of the kinase.

Mutations in leucine-rich repeat kinase 2 (*LRRK2*) are one of the most common causes of familial Parkinson’s disease (PD) (1, 2). LRRK2 is a large multidomain protein. Its N-terminal half contains armadillo, ankyrin, and leucine-rich repeats, while its C-terminal catalytic half contains GTPase (Ras of complex, or ROC), C-terminal of ROC (COR-A and COR-B), kinase, and WD40 domains (Fig. 1*A*). LRRK2 forms dimers at two interfaces: between its WD40 domains and between its COR-B domains (3, 4, 5); these interfaces are important for LRRK2 to form filaments on microtubules, which is enhanced by high expression levels of LRRK2, in the presence of LRRK2 type-I kinase inhibitors, and with overexpression of most PD variants of LRRK2 (3, 6, 7, 8). Most PD-linked variants of LRRK2 also increase its kinase activity (9, 10, 11, 12). Thus, small molecules that inhibit LRRK2’s kinase activity are promising drug candidates for PD treatment (13).

**Figure 1:**
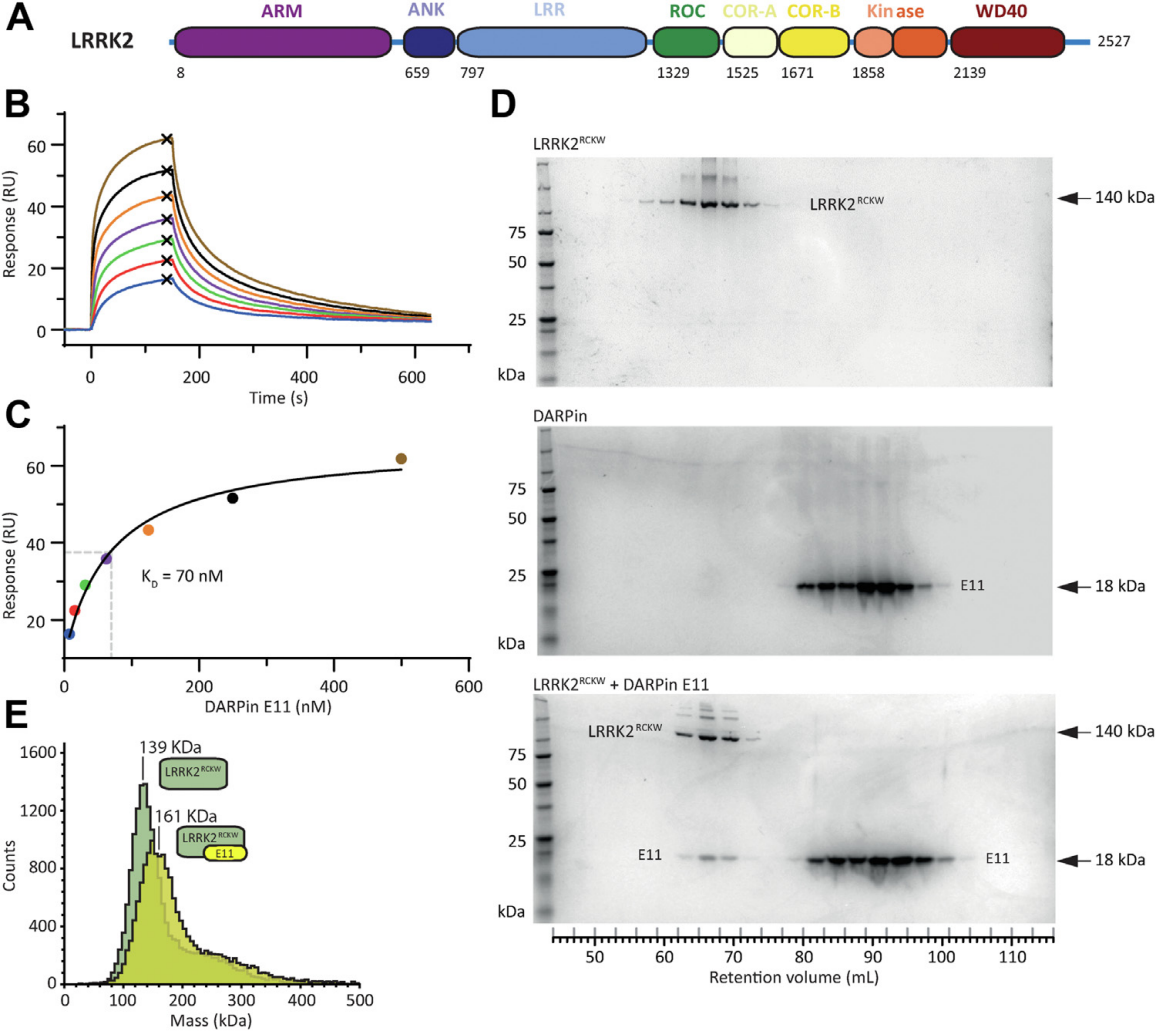
**DARPin E11 binds to LRRK2**^**RCKW**^**with high affinity**. *A*, domain architecture of the LRRK2 protein. The same color scheme is used in all figures. The first residue of each domain is indicated. *B* and *C*, surface plasmon resonance analysis of DARPin E11 binding to LRRK2^RCKW^. Recombinant LRRK2^RCKW^ was immobilized on a sensor chip and varying concentrations of DARPin E11 were added in the mobile phase. Sensograms are shown (no fit or errors were applied) (*B*) and the plateau values from the dose-response curves (*C*) are shown and fit to the Langmuir equation according to the least square method. A dissociation constant (K_D_) of 70 nM ± 11 nM (SEM) was determined. *D*, the LRRK2^RCKW^:E11 complex was subjected to size exclusion chromatography (SEC). Elution fractions were analyzed by SDS PAGE and visualized by Coomassie staining. The co-elution of LRRK2^RCKW^ and DARPin E11 showed that the complexes were stable during the SEC run. Molecular weight markers are noted on the left and the molecular weights of LRRK2^RCKW^ and DARPin E11 on the right. *E*, analysis of the LRRK2^RCKW^:E11 complex by mass photometry. The molecular weight obtained from the yellow peak corresponds to that of a 1:1 LRRK2^RCKW^:E11 complex. The tabular data for this figure can be found at: https://doi.org/10.5281/zenodo.10471514. DARPin, designed ankyrin-repeat protein; LRRK2, leucine rich repeat kinase 2.

Given the complexity of LRRK2 regulation and signaling, it is necessary to study these processes in cellular environments. Small molecule and protein-based binders have been developed for this purpose. The most important of these in terms of therapeutic development are the type-I kinase inhibitors that bind to the active-like state of LRRK2. Several type-I inhibitors have been developed, including MLi-2 (14) and DNL201 (developed as GNE0877) (15). DNL151 (renamed BIIB122), which is related to DNL201, has entered phase 2b clinical trials (clinicaltrials.gov identifier NCT05348785). In contrast, there are currently no published LRRK2-selective type-II inhibitors that target the LRRK2 inactive state. An example of a high-affinity, but not selective type-II inhibitor for LRRK2 is rebastinib (16). In addition to kinase inhibitors, other LRRK2 tools have been developed. These include two proteolysis targeting chimeras (PROTAC) called XL01126 (17) and JH-XII-03-02 (18), a small molecule that binds to its ROC GTPase domain called FX2149 (19), and nanobodies (20, 21).

In addition to nanobodies, designed ankyrin repeat proteins (DARPins) are a class of protein-based binders that have been used in research, diagnostics and therapy (22). DARPins are inspired by the naturally occurring ankyrin repeat (AR) domains. They comprise two to three ARs that each contain randomized regions (7 randomized residues per repeat), flanked by capping repeats with a hydrophilic surface, and newer versions of the libraries also contain randomized loops and randomized regions in the capping repeats (22, 23). The theoretical diversity far exceeds the amount of DNA encoding it, but with ribosome display (24, 25), libraries of 10^12^ are typically used for selecting specific binders, limited only by the number of ribosomes in the reaction mix (22). Binders to several hundred different targets have been generated. As specific binding proteins, DARPins have similar applications as antibodies but feature several advantages: they are smaller in size, are very stable and rigid, can be produced in *Escherichia coli*, harbor no disulfide bonds, and can thus be used in reducing cytoplasm and are easier to engineer to different formats. Some DARPins have entered clinical trials (ClinicalTrials.gov identifiers NCT02194426, NCT03084926, NCT03136653, NCT04049903, NCT04834856, NCT05098405, NCT05673057, NCT03335852, NCT02859766, NCT02462928, NCT02462486, NCT02186119, and NCT02181517), and they have been used as conformation-specific sensors in living cells (26, 27).

DARPins have also been used to modulate sample properties in cryo-EM applications. DARPins can be designed to selectively bind to and stabilize specific conformations or states of the target molecules (27). They have been useful for studying conformational changes or for helping to understand the molecular determinants of protein activation and autoinhibition (28). Currently, proteins smaller than about 50 kDa present a challenge for structural elucidation by cryo-EM. Recent reports have shown that small proteins can be visualized attached to a large, symmetric base or cage *via* a DARPin (29, 30, 31). The additional molecular mass and features contributed by the DARPin can also improve the signal-to-noise ratio and facilitate the determination of high-resolution structures. Finally, the adoption of a limited number of orientations on the grid by a target molecule, resulting from the interaction with the air-water interface, remains the most common and significant challenge in cryo-EM. DARPins could help increase the number of orientations adopted by a target molecule by changing its surface properties.

Here, we describe the identification and characterization of a DARPin (E11) that binds to LRRK2 with high affinity. DARPin E11 did not affect the kinase activity of a truncated LRRK2 construct *in vitro*. Surprisingly, however, it inhibited full-length LRRK2’s ability to phosphorylate Rab8A, one of LRRK2’s physiological substrates, when expressed in cells. Expressing DARPin E11 in cells also prevented the formation of microtubule-associated LRRK2 filaments, which are mediated by a WD40-WD40 interaction that overlaps with the E11 binding site on the WD40 domain. Our cryo-EM analysis of the LRRK2:E11 complex indicated that addition of the DARPin increased the number of orientations adopted by LRRK2 on cryo-EM grids, leading to an improved cryo-EM structure. Thus, DARPin E11 is a new tool with potential applications in structural studies of LRRK2:ligand complexes, functional studies of LRRK2 in cells, and the design of LRRK2 inhibitors that target its WD40 domain rather than its kinase domain.

## Results

### Identification of LRRK2-specific DARPins

To identify LRRK2-specific DARPins, we started with a library of N2C and N3C DARPins with randomized loops and capping repeats. For LRRK2, we used the well-characterized C-terminal catalytic half of LRRK2 that contains its ROC GTPase, COR-A and COR-B, kinase, and WD40 domains that we refer to as LRRK2^RCKW^ (Fig. 1*A*) (3). We then used ribosome display with immobilized LRRK2^RCKW^ protein to enrich the DARPin pool for those that bound LRRK2 (24). After four rounds of selection, 380 of the obtained DARPins were expressed in *E. coli* and probed in a homogeneous time-resolved fluorescence (HTRF) assay. Twenty of these DARPins produced promising results *via* HTRF, but most of them tended to self-associate, making further biochemical analysis challenging. One DARPin, 008-807-2362-H9 (renamed which we named "E11"), was monomeric in solution and exhibited high affinity binding to LRRK2^RCKW^ leading us to focus on it for further characterization.

### Biophysical characterization of the LRRK2^RCKW^:E11 complex

To characterize DARPin E11, we first performed surface plasmon resonance (SPR) experiments, applying DARPin E11 solutions at multiple concentrations to obtain association and dissociation kinetics (Fig. 1, *B* and *C*). The plateau values were fitted to a Langmuir model, and the K_D_ value was calculated to be 70 nM. We also probed the LRRK2^RCKW^:E11 interaction by bio-layer interferometry. Biotinylated LRRK2^RCKW^ was immobilized on the biosensors, and multiple concentrations of DARPin E11 were used. The dose-response curve was fitted to a Langmuir model, resulting in a K_D_ value of 11 nM (Fig. S1*A*). Together, our data indicate that DARPin E11 bound tightly to LRRK2^RCKW^ with K_D_ values in the low nanomolar range. The dissociation off-rate of the LRRK2^RCKW^:E11 complex (k_off_ in SPR ∼0.005 s^−1^) suggested that the complex is long-lived; this was supported by its stability in size-exclusion chromatography (SEC) experiments (Figs. 1*D* and S1*B*). Finally, we compared LRRK2^RCKW^ alone to the LRRK2^RCKW^:E11 complex using mass photometry, where samples are at equilibrium. As expected, LRRK2^RCKW^ alone was mainly monomeric with a minor portion of dimers. The addition of a 2.5-fold excess DARPin E11 shifted the entire histogram to higher masses to a mass that corresponded to the addition of a DARPin molecule (Fig. 1*E*). These results demonstrate the reversible formation of stable complexes with low nanomolar affinity between LRRK2^RCKW^ and DARPin E11.

### Cryo-EM structure of LRRK2^RCKW^ bound to DARPin E11

**Figure 2:**
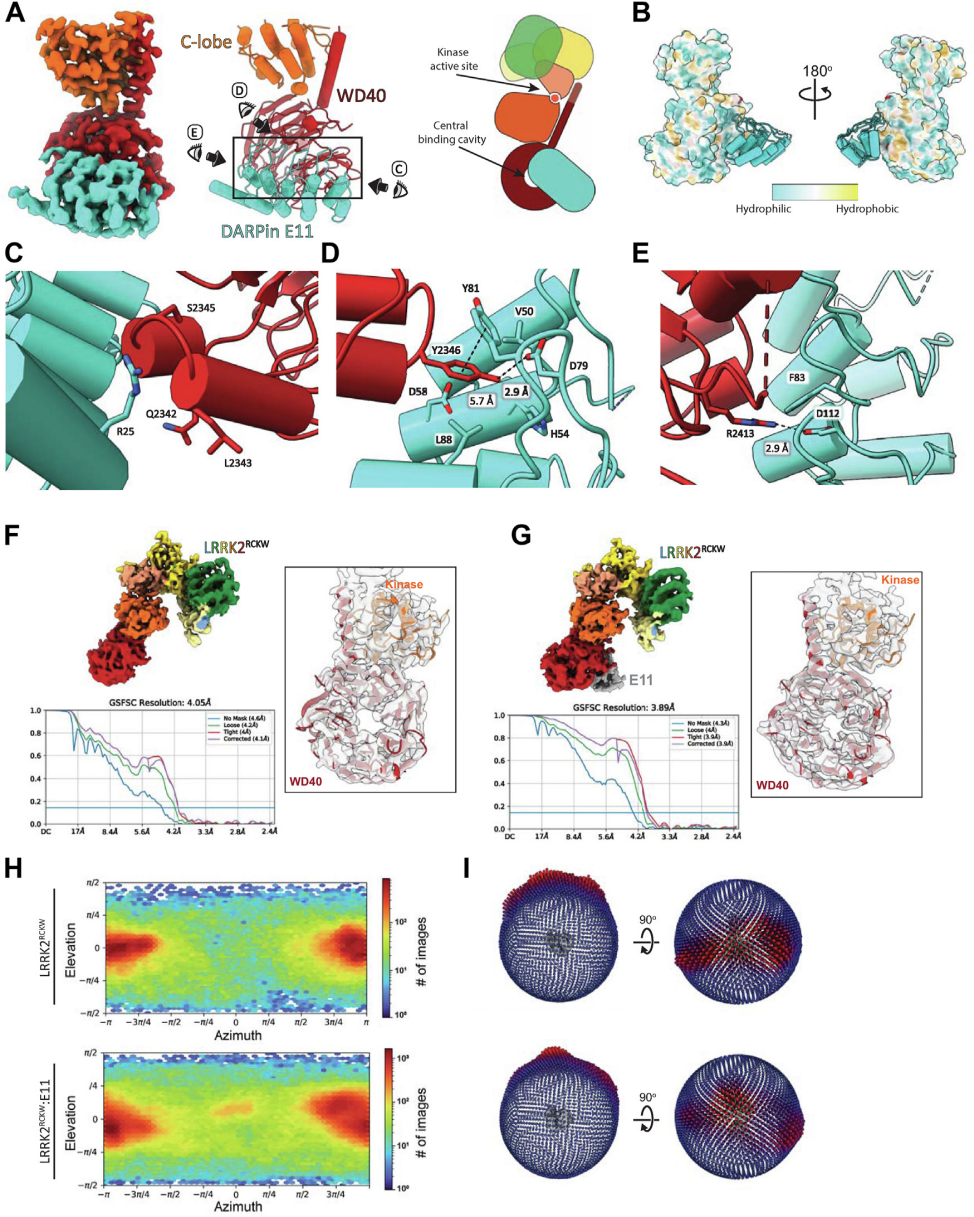
**Cryo-EM structure of the LRRK2**^**RCKW**^**:DARPin E11 complex.***A*, Cryo-EM map (*left*), model (*middle*), and schematic representation (*right*) of LRRK2^RCKW^ bound to DARPin E11. Because of the focused refinement strategy used to maximize the resolution of the DARPin-WD40 part of the structure, only the kinase C-lobe and the WD40 domain are seen in this map. E11 binds to one of the faces of the WD40 domain, opposite the kinase active site, next to the central WD40 cavity. The *eyes* and *arrows* surrounding the *rectangle* on the model indicate the direction of the views shown in panels (*C*–*E*). *B*, surface representation of hydrophobicity of LRRK2^KW^ bound to DARPin E11, displaying the latter as a model. The most polar residues are shown in *blue*, and the most hydrophobic residues are shown in *gold*. *C*–*E*, close-up views of the binding interface. Key residues for the interaction are highlighted. *F* and *G*, Cryo-EM maps, FSC plots, and model-to-map fits for LRRK2^RCKW^ alone or bound to DARPin E11. *H* and *I*, plots of Euler angle distribution generated in cryoSPARC (*left*) or Relion (*right*) for LRRK2^RCKW^ alone (*top*) or bound to DARPin E11 (*bottom*). Addition of E11 increased the number of orientations adopted by LRRK2^RCKW^ on the cryo-EM grids. DARPin, designed ankyrin-repeat protein; FSC, fourier shell correlation; LRRK2, leucine rich repeat kinase 2.

Next, we wanted to determine the binding mode between DARPin E11 and LRRK2. To do this, we prepared samples for analysis by cryo-EM by mixing LRRK2^RCKW^, GDP/Mg^2+^, a cofactor of LRRK2’s Ras-like GTPase domain (ROC) and DARPin E11. The workflow for data collection and analysis is summarized in Fig. S2. The resulting cryo-EM map and model showed that DARPin E11 bound to the LRRK2 WD40 domain. The binding interface between DARPin E11 and LRRK2^RCKW^ is located on one of the faces of the WD40 domain, adjacent to its central cavity and opposite the kinase active site (Fig. 2*A*). Two insertions connecting neighboring blades of the WD40 ß-propeller (which contains seven blades) are particularly prominent in the binding interface: the helix-loop-helix motif connecting blades 4 and 5 (residues 2339–2351) and the partially disordered insertion connecting blades 5 and 6 (residues 2388–2414). The area of the binding interface, calculated using the proteins, interfaces, structures and assemblies (PISA) server, is 877 Å^2^, a value that is similar to what has been reported for other DARPin complexes (32, 33). In addition to hydrophobic interactions (Fig. 2*B*), several key residues in LRRK2^RCKW^ and DARPin E11 were identified that form polar interactions. (i) The backbone carbonyls from LRRK2^RCKW^ residues Gln2342, Leu2343, and Ser2345 form an interaction network with residue Arg25 from DARPin E11 (Fig. 2*C*). (ii) Several DARPin E11 residues, including Val50, His54, Asp58, Asp79, Tyr81, and Leu88, form a deep pocket that accommodates the side chain of the LRRK2^RCKW^ residue Tyr2346. The Asp79 side chain resides in the back of this pocket within distance to form an electrostatic interaction with the hydroxyl group of Tyr2346. Furthermore, Tyr81 and Tyr2346 show π-π stacking, highlighting the importance of residue Tyr2346 in DARPin E11 binding (Fig. 2*D*). (iii) Another key residue for the interaction is LRRK2^RCKW^ Arg2413. Its guanidine group is close to the DARPin E11 residues Phe83, Asp112, and His113, the latter allowing for electrostatic interactions (Fig. 2*E*). As expected, on the DARPin E11 side most interacting residues are situated in the randomized regions of the core helices. Interestingly, several interacting residues including Arg25 and Asp58 are located in the core helices (Fig. 2*C*).

### DARPin E11 reduces preferred orientation on cryo-EM grids

The DARPin E11 proved to be useful in a way we had not anticipated. When we determined the LRRK2^RCKW^:E11 structure, we noticed that the cryo-EM map of the complex showed better density for LRRK2^RCKW^ than what we had observed in the map of LRRK2^RCKW^ solved in the absence of E11 (Fig. 2, *F* and *G*). The reason for this improvement appeared to be a better distribution of particle views in the presence of DARPin E11 (Fig. 2, *H* and *I*), although we cannot rule out an effect from the additional mass and features contributed by the DARPin. Given that both LRRK2^FL^ and LRRK2^RCKW^ have shown strong preferred orientations in the past (3, 4), DARPin E11, and LRRK2-specific DARPins in general, have the potential to significantly improve the LRRK2 structures that can be obtained using cryo-EM. In fact, we used E11 in our recent structures of LRRK2 bound to type 1 and type 2 kinase inhibitors (34).

### DARPin E11 does not alter the kinase activity of LRRK2^RCKW^*in vitro*

**Figure 3:**
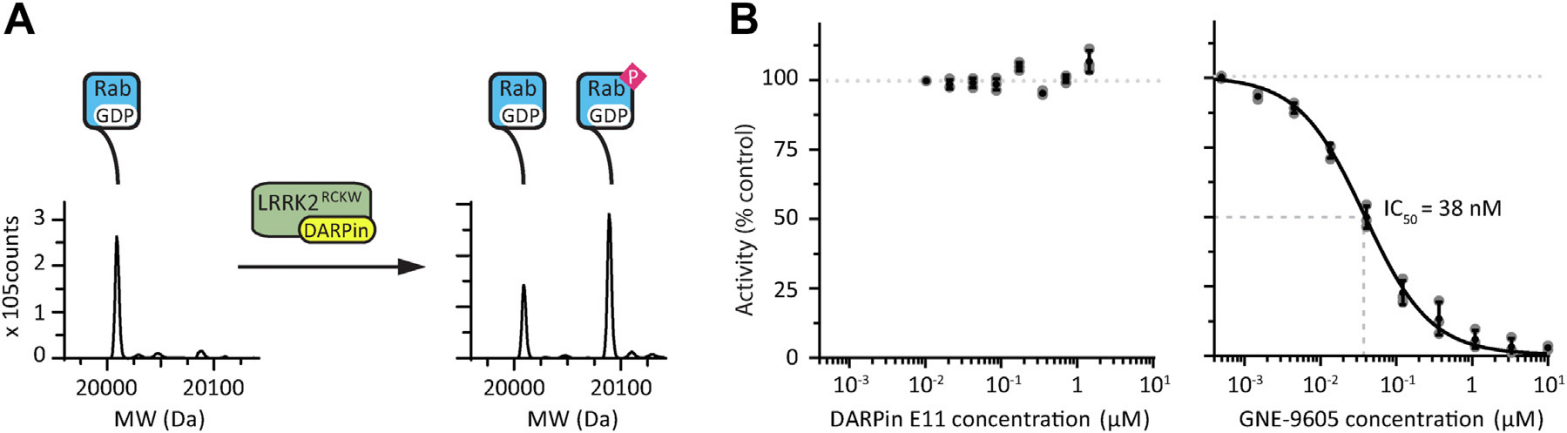
**DARPin E11 does not affect the kinase activity of LRRK2**^**RCKW**^***in vitro***. *A*, *in vitro* assay measuring the level of Rab8A phosphorylation by LRRK2^RCKW^. Rab8A and LRRK2^RCKW^ were incubated in the presence of ATP and kinase activity was monitored by measuring the levels of substrate (Rab8A) and product (pRab8A) by mass spectrometry. *B*, Kinase activity of LRRK2^RCKW^ in the presence of either DARPin E11 (*left*) or the LRRK2-specific kinase inhibitor GNE-9605 (*right*). The IC_50_ was 38 nM ± 3 nM (SD). Plot points represent three technical replicates. DARPin, designed ankyrin-repeat protein; LRRK2, leucine rich repeat kinase 2.

We next sought to understand the functional implications of the LRRK2:DARPin E11 interaction. A subset of Rab GTPases, which mark specific membrane compartments in cells, are known substrates of LRRK2 (11, 35). Thus, we determined if DARPin E11 binding to LRRK2^RCKW^ impacted its kinase activity. To do this, we performed *in vitro* kinase assays with LRRK2^RCKW^ and its substrate Rab8A in the presence of varying concentrations of DARPin E11 (Fig. 3*A*). The ratio between the nonphosphorylated Rab8A and phosphorylated Rab8A (pRab8A) was determined using electrospray time-of-flight mass spectrometry (MS). These assays revealed that even at the saturating concentration of 1 μM, DARPin E11 had no effect on LRRK2^RCKW^ kinase activity (Fig. 3*B*).

**Figure 4:**
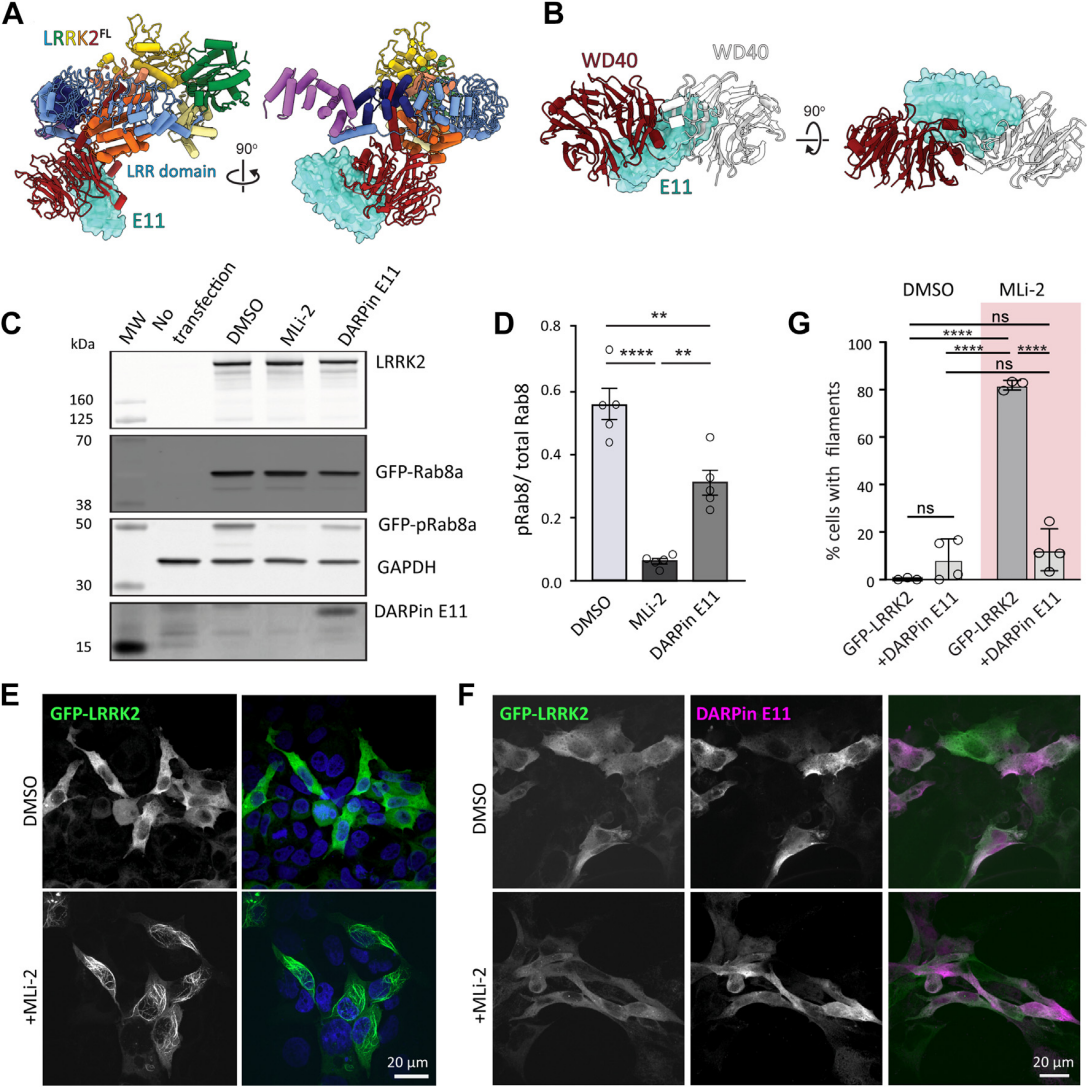
**DARPin E11 disrupts LRRK2**^**FL**^**filament formation and decreases Rab8 phosphorylationin cells.***A*, modeling of E11 bound to the autoinhibited conformation of LRRK2^FL^ shows no steric clashes between E11 and LRRK2. *B*, the binding site of DARPin E11 on the WD40 domain overlaps with the WD40:WD40 dimerization interface that is involved in the formation of microtubule associated LRRK2 filaments. *C*, Rab8 phosphorylation in 293T cells overexpressing LRRK2^FL^ and GFP-Rab8, with or without DARPin E11-3xFLAG. 293T cells were transiently cotransfected with LRRK2^FL^ and GFP-Rab8 or LRRK2^FL^, GFP-Rab8, and DARPin E11-3xFLAG for 48 h. Cells transfected only with LRRK2^FL^ and GFP-Rab8 were treated with DMSO or 2 μM MLi-2 for 1 h. Cells were lysed and immunoblotted for phospho-Rab8 (pT72), total GFP-Rab8, total LRRK2, DARPin E11-3xFLAG, and GAPDH. MW; molecular weight marker. *D*, quantification from five Western blots plotting the ratio of GFP-pRab8 to total GFP-Rab8. Plot points represent four technical replicates. Statistics were generated in GraphPad using a one-way ANOVA analysis with a Tukey’s multiple comparison of means. ∗∗∗∗*p* <0.0001 DMSO and MLi-2, ∗∗*p* = 0.0013 DMSO and DARPin E11, ∗∗*p*= 0.0012 MLi-2 and DARPin E11. *E* and *F*, representative images of 293T cells expressing either GFP-LRRK2^FL^ (*E*) or GFP-LRRK2^FL^ and DARPin E11 (*F*), treated with DMSO or MLi-2 for 2 h. *G*, quantification of the percent cells (mean ± sd) with GFP-LRRK2^FL^ filaments in the presence or absence or DARPin E11. Each data point on the graph represents a technical replicate. Three or four independent replicates were done per condition, with each replicate containing 48 to 140 cells. Statistics were generated using an one Way ANOVA with a Tuckey’s multiple comparison of means. ∗∗∗∗ *p* < 0.0001. ns, not significant. The tabular data for this figure can also be accessed at https://doi.org/10.5281/zenodo.10530220. DARPin, designed ankyrin-repeat protein; DMSO, dimethylsulfoxide; LRRK2, leucine rich repeat kinase 2.

Full-length LRRK2 (LRRK2^FL^) has been reported to adopt an autoinhibited conformation (4). The mechanism of autoinhibition has been proposed to be mediated by the N-terminal LRR domain folding over the kinase active site, a structural arrangement that partially blocks the substrate binding site of LRRK2. Folding of the LRR domain over the kinase active site is stabilized by interactions with the WD40 domain and the C-terminal helix of LRRK2. Therefore, we hypothesized that DARPin E11 interacting with the WD40 domain might interfere with the autoinhibited conformation. However, the overlay of the autoinhibited LRRK2^FL^ model (4) and our LRRK2^RCKW^:E11 model showed that this was not the case, as DARPin E11 could bind to the WD40 domain without introducing clashes with the LRR (Fig. 4*A*). However, the DARPin E11 binding site on LRRK2^RCKW^ did overlap with the site of WD40:WD40 dimerization (Fig. 4*B*).

### DARPin E11 decreases LRRK2 kinase activity in cells

Our experiments with LRRK2^RCKW^ showed that DARPin E11 did not affect LRRK2 kinase activity *in vitro*. Next, we wanted to determine if DARPin E11 affected LRRK2 kinase activity in cells. We transfected 293T cells with full-length untagged LRRK2 and GFP-Rab8A with or without cotransfection of DARPin E11-3xFLAG and monitored the phosphorylation of GFP-Rab8A using a phospho-specific Rab8A antibody. Strikingly, in contrast to what we observed *in vitro* using the N-terminally truncated LRRK2^RCKW^ construct, the presence of DARPin E11 significantly reduced LRRK2^FL^ kinase activity in cells compared to the dimethylsulfoxide (DMSO) control (Fig. 4, *C* and *D*).

### DARPin E11 disrupts LRRK2 microtubule association

LRRK2 oligomerizes and interacts with microtubules in cells under some circumstances (7). These LRRK2 filaments have been observed by cryo-electron tomography in cells for LRRK2^FL^ (8) and *in vitro* for LRRK2^RCKW^ (36). The tendency of LRRK2 to form filaments is increased by most LRRK2 PD mutations (7) and by the presence of type-I kinase inhibitors that stabilize the LRRK2 active conformation (3, 6, 7, 16). The LRRK2 filaments are formed by LRRK2 dimers, mediated by a COR-B:COR-B interface, which interacts through a WD40:WD40 interface (3, 8, 36). This interface is best resolved in a crystal structure of WD40 dimers (5). The overlay with our LRRK2^RCKW^:E11 complex showed that the presence of DARPin E11 was incompatible with the formation of the WD40:WD40 dimer (Fig. 4*A*). Thus, we hypothesized that DARPin E11 would prevent the formation of LRRK2 filaments in cells. To test this, we coexpressed DARPin E11 and GFP-LRRK2^FL^ in 293T cells and treated the cells with the LRRK2-specific type-I inhibitor MLi-2. Like other LRRK2 type-1 inhibitors, MLi-2 induces LRRK2 filament formation on microtubules in cells (3, 6, 7, 16). As expected, treatment of cells with MLi-2 induced LRRK2 filament formation (Fig. 4, *E* and *F*). While LRRK2 filaments formed in over 80% of control cells transfected with GFP-LRRK2^FL^ and treated with MLi-2, cells coexpressing DARPin E11 did not form filaments in the presence of MLi-2, suggesting that DARPin E11 disrupts WD40-dependent LRRK2 dimerization and its association with microtubules (Fig. 4*G*).

## Discussion

Here, we described the development of a LRRK2-specific DARPin, DARPin E11. The development of this small protein-based probe for LRRK2 adds an important reagent to the growing toolbox to explore LRRK2 function in health and disease. We showed that DARPin E11 binds to the WD40 domain of LRRK2 with high affinity.

Unexpectedly, we found that binding of DARPin E11 to LRRK2’s WD40 domain decreased preferred orientations on cryo-EM grids, allowing us to solve a cryo-EM structure with improved resolution. Thus, DARPin E11, and potentially other LRRK2-specific DARPins, will facilitate future cryo-EM studies of LRRK2. The higher resolutions obtained in the presence of DARPin E11 were particularly useful in our recent work on structures of LRRK2 in complex with kinase inhibitors (34).

Our studies of DARPin E11 revealed that this affinity reagent can be used to study intramolecular and intermolecular interactions modulating LRRK2 functions in cells. For instance, we showed that DARPin E11 disrupted the ability of LRRK2 to form microtubule-associated filaments, which form in the presence of the LRRK2 kinase inhibitor MLi-2 and are enhanced by many PD mutations (7). Thus, DARPin E11 could be used as a tunable reagent in cells to prevent the formation of the WD40-WD40 interface. In the future, this reagent could be used to study endogenous LRRK2 in PD relevant cell types.

In terms of LRRK2’s cellular function, our most intriguing observation was that even though DARPin E11 had no effect on the kinase activity of truncated LRRK2^RCKW^ *in vitro*, its expression in cells led to reduced phosphorylation of Rab8A, one of LRRK2’s physiological substrates. This suggests that DARPin E11 could be competing with accessory factors that interact with the WD40 domain to control LRRK2’s subcellular localization, orientation, and/or conformation. This idea is consistent with the properties of a rare LRRK2 variant (V2390M) identified in a male PD patient from Spain (37). This variant showed reduced phosphorylation of Rab10 in cells (38). While the small valine side chain (V2390) is accommodated well in our structure of the LRRK2^RCKW^:E11 complex, the bulkier methionine side chain has the potential to disrupt, or at least weaken the binding of E11 to LRRK2’s WD40 domain. We speculate that DARPin E11 and the V2390M variant may be disrupting a binding site for cellular LRRK2 regulators. Alternatively, it is possible that the N-terminal repeats play a role in the inhibitory effect seen with E11 in cells. However, our current structural understanding of LRRK2 and of the complex between E11 and LRRK2^RCKW^ does not provide an explanation for such an effect. Thus, at this time the most likely explanation for E11’s effect on LRRK2’s kinase activity is that it competes for a binding site on LRRK2 with an activating factor. The identity of such a factor and how it modulates LRRK2 kinase activity remains unknown, but DARPin E11 is a tool that could lead to its discovery.

The observations summarized above also suggest a new approach to design small-molecule inhibitors of LRRK2’s kinase activity in cells by targeting its WD40 domain rather than its kinase: small-molecule libraries could be screened for binders that disrupt the interaction between LRRK2’s WD40 domain and DARPin E11. The identified small-molecule binders would serve as starting points for the development of high-affinity binders of the LRRK2 WD40 domain that may be potent inhibitors of LRRK2 in cells and could be further developed as therapeutics for PD.

## Experimental procedures

### Production of the biotinylated LRRK2^RCKW^ protein

The DNA coding for the LRRK2 residues 1327 to 2527 (OHu107800 from Genscript, RRID:SCR_002891) was PCR-amplified using the forward primer TACTTCCAATCCATGAAAAAGGCTGTGCCTTATAACCGA and the reverse primer TATCCACCTTTACTGCTCTCAACAGATGTTCGTCTCATTTTTTCA. The T4 polymerase-treated amplicon was inserted into the transfer vector pFB-Bio5 (SGC) by ligation-independent cloning. The resulting plasmid was utilized for the generation of recombinant Baculoviruses according to the Bac-to-Bac expression system protocol (Invitrogen, 10359016). Exponentially growing Sf9 cells (2 × 10^6^ cells/ml (RRID:CVCL_0549) in Lonza Insect-XPRESS medium (Lonza RRID:SCR_000377, BELN12–730) supplemented with 0.1 mM biotin (Roth, 3822.4) were infected with a high-titer Baculovirus suspension. After 66 h of incubation (27 °C and 90 rpm), cells were harvested by centrifugation. The expressed protein construct contained an N-terminal His_6_-tag, cleavable with tobacco etch virus (TEV) protease. For LRRK2^RCKW^ purification, the pelleted Sf9 cells were washed with PBS, resuspended in lysis buffer (50 mM Hepes pH 7.4, 500 mM NaCl, 20 mM imidazole, 0.5 mM TCEP, 5% glycerol), and lysed by sonication. The lysate was cleared by centrifugation and loaded onto a Ni NTA column (Qiagen, 30250). After vigorous rinsing with lysis buffer the His_6_-tagged protein was eluted in lysis buffer containing 300 mM imidazole. The eluate was treated with TEV protease to cleave the His_6_-tag and dialyzed overnight in storage buffer (20 mM Hepes pH 7.4, 150 mM NaCl, 0.5 mM TCEP, and 5% glycerol) to reduce the imidazole concentration. Contaminating proteins, uncleaved LRRK2^RCKW^ protein and TEV protease were removed with another Ni NTA step. Finally, LRRK2^RCKW^ was concentrated and subjected to gel filtration in storage buffer using an AKTAxpress system combined with an S200 gel filtration column. The elution volume 69.3 ml indicated the protein to be monomeric in solution. The final yield as calculated from UV absorbance was 0.6 mg biotinylated LRRK2^RCKW^/L insect cell medium. Our current protocol for this assay can also be found at (dx.doi.org/10.17504/protocols.io.8epv5xpz4g1b/v1).

### Selection of DARPins

DARPins were selected as described previously (Dreier and Plückthun, 2012; Plückthun, 2012). In brief, DARPins were selected over four rounds using a KingFisher Flex MTP96 well platform, and 380 single DARPin clones were screened for binding to LRRK2^RCKW^ using HTRF performed according to a previously established protocol (24, 27). From the identified hits, 32 single clones were chosen for sequencing, and 20 unique sequences were identified. Here, we analyzed one of these unique clones, DARPin E11, whose protein sequence is given below:

MRGSHHHHHHHHGSDLGKKLLEAARAGQDDEVRILMANGADVNATDEAGVTPLHLAADSGHLEIVEVLLKTGADVNAWDHYGFTPLHLAAHVGHLEIVEVLLKAGADVNAQDHAGWTPLHLAALYGHLEIVEVLLKHGADVNAQDMWGETPFDLAIDNGNEDIAEVLQKAAKLNDYKDDDDK.

### Expression and purification of DARPin E11 and Rab8A

DARPin E11 was cloned into a pQE30 vector (Qiagen). The expression construct included an N-terminal 8× His tag and a C-terminal 3x-FLAG tag. Rab8A was cloned into a pET-28a vector (Novagen). The expression construct comprised Rab8A residues G6-K176 and an N-terminal TEV-cleavable 6× His tag. The plasmids were transformed into *E. coli* Rosetta cells. Cells were grown at 37 °C with shaking until the *A*_600_ reached 1.0. Then, the temperature was reduced to 18 °C and protein expression was induced by adding 0.5 mM IPTG. After 18 h, the cells were harvested by centrifugation and resuspended in lysis buffer (50 mM Hepes pH 7.4, 500 mM NaCl, 20 mM imidazole, 0.5 mM TCEP, and 5% glycerol) prior to lysis by sonication. The lysate was cleared by centrifugation and loaded onto preequilibrated Ni-NTA Sepharose beads (GE HealthCare). The beads were washed with 50 column volumes of lysis buffer. The respective protein was eluted in lysis buffer supplemented with 300 mM imidazole. The eluate was concentrated to 5 ml and subjected to SEC using an S200 16/200 column (GE HealthCare) in SEC buffer (20 mM Hepes pH 7.4, 150 mM NaCl, 0.5 mM TCEP, and 5% glycerol).

### Determination of DARPin affinities by SPR

The SPR analysis was performed on a Biacore T200 (Cytiva Life Sciences). Subsequently, 850 RU of biotinylated LRRK2^RCKW^ was loaded onto a Series S CM5 chip coated with streptavidin. The chip was equilibrated with running buffer containing 20 mM Hepes pH7.4, 150 mM NaCl, 0.5 mM TCEP, 20 μM GDP, and 2.5 mM MgCl_2_. A titration was performed for DARPin E11. The DARPin was allowed to bind over the surface at a flow rate of 30 μl/min for 150 or 180 s and disassociation was followed for 480 s. The sensograms were double-reference subtracted and analyzed by a 1 + 1 kinetic model and a 1 + 1 Langmuir adsorption isotherm model for DARPin E11.

### Size-exclusion chromatography

Analyzed samples contained LRRK2^RCKW^ (final concentration 10 μM), DARPin E11 (200 μM), or a mixture of both proteins. Two milliliters of each sample was subjected to SEC on an ÄKTA Xpress system combined with an S200 gel filtration column (running buffer 20 mM Hepes pH 7.4, 500 mM NaCl, 2.5 mM MgCl_2_, 20 μM GDP, 0.5 mM TCEP, and 0.5% glycerol). During the SEC run, the protein fold and potential protein complexes remained intact. The flowthrough was collected in 3-mL fractions. To analyze the protein content of each fraction, the proteins were denatured, separated according to their size by SDS PAGE, and the gels stained with Coomassie.

### Determination of DARPin affinities by bio-layer interferometry

The measurements were performed using an Octet Red96 (FortéBio) system at 25 °C in a buffered solution containing 50 mM Hepes pH 8.0, 150 mM NaCl, 10 mM MgCl_2_, 5% glycerol, 0.1% bovine serum albumin (BSA), 0.05% Tween 20, and 500 μM GDP. Binding of DARPins to LRRK2^RCKW^ was measured using biotinylated LRRK2^RCKW^ protein loaded onto streptavidin-coated (SA) biosensors. The protein was immobilized to the sensors from a fixed LRRK2^RCKW^ concentration of 5 μg/ml, and binding curves were measured by varying concentrations of DARPin E11 from 15 to 1000 nM. The association/dissociation traces were fitted using the global option (implemented in the FortéBio Analysis Software (https://www.sartorius.com/en/products/protein-analysis/octet-bli-detection/octet-systems-software, RRID:SCR_023267). The resulting R_eq_ values were subsequently plotted against the DARPin concentration and used to derive the K_D_ values from the corresponding dose-response curves by fitting on a Langmuir model. Dose-response curves were measured in triplicates. Final figures were generated using GraphPad Prism7 (http://www.graphpad.com/, RRID:SCR_002798).

### Mass photometry analysis

Mass photometry data for DARPin E11 was acquired on a Refeyn Two MP mass photometer with the Refeyn Acquire MP Software 2023.1.1 (https://www.refeyn.com/twomp-mass-photometer) The measurements were carried out in reusable gaskets (Grace Bio Labs, CW-50R-1.0 50-3DIA x1 mm) on high precision cover slips which had been cleaned with isopropanol, 70% ethanol, and Milli-Q water. Subsequently, 20 μl buffer (20 mM Hepes pH 7.4, 150 mM NaCl, 2.5 mM MgCl_2_, 5% glycerol, 20 μM GDP, and 0.5 mM TCEP) was added to the gaskets. After focusing, 10 μl of the buffer drop was exchanged for the protein solution to be measured. For landing events, 2997 image frames were acquired during 60 s measurement time at 499.5 Hz. All data were analyzed with the RefeynDiscover MP Software 2023.1.2 (https://www.refeyn.com/twomp-mass-photometer). Ratiometric contrast values were converted into molecular mass using a standard mass calibration (using BSA, beta-amylase from sweet potato, and bovine thyroglobulin as reference).

### Purification of LRRK2^RCKW^ for Cryo-EM

Sf9 cells expressing His_6_-Z-TEV-LRRK2^RCKW^ were washed with PBS, resuspended in lysis buffer (50 mM Hepes pH 7.4, 500 mM NaCl, 20 mM imidazole, 0.5 mM TCEP, 5% glycerol, 5 mM MgCl_2_, and 20 μM GDP) and lysed by homogenization. The supernatant was cleared by centrifugation and loaded onto a Ni-NTA (Qiagen) column. After rinsing with lysis buffer, the His_6_-Z-tagged protein was eluted in lysis buffer containing 300 mM imidazole. The eluate was then diluted to 250 mM NaCl with a dilution buffer (50 mM Hepes pH 7.4, 0.5 mM TCEP, 5% glycerol, 5 mM MgCl_2_, and 20 μM GDP) and loaded onto an SP sepharose column. His_6_-Z-TEV-LRRK2^RCKW^ was eluted with a gradient from 250 mM to 2.5 M NaCl in dilution buffer and treated with TEV protease overnight to cleave the His_6_-Z-tag. Contaminating proteins, the cleaved tag, uncleaved protein, and TEV protease were removed by another combined SP sepharose Ni-NTA step. Finally, LRRK2^RCKW^ was concentrated and subjected to gel filtration in 20 mM Hepes pH 7.4, 700 mM NaCl, 0.5 mM TCEP, 5% glycerol, 2.5 mM MgCl_2_, and 20 μM GDP using an AKTA Xpress system combined with an S200 gel filtration column. The final yield, as calculated from UV absorbance, was 2.1 mg of LRRK2^RCKW^ per liter of insect cell medium. Our current protocol for this purification can be found at (dx.doi.org/10.17504/protocols.io.81wgb6693lpk/v1).

### Preparation of LRRK2^RCKW^:E11 complex for structural studies

LRRK2^RCKW^ was exchanged into buffer A (20 mM Hepes pH 7.4, 150 mM NaCl, 2.5 mM MgCl_2_, 5% glycerol, 20 μM GDP, and 0.5 mM TCEP). DARPin E11 was incubated with LRRK2^RCKW^ in a ratio of 1:1.25 for 10 min at room temperature (RT) and then placed at 4 °C and incubated for 15 min. After that, the sample was diluted with cold buffer A to a final LRRK2^RCKW^ concentration of 6 μM. The sample was used to make cryo-EM grids immediately after dilution. Our current protocol can be found at (dx.doi.org/10.17504/protocols.io.j8nlkowxxv5r/v1).

### Cryo-EM sample preparation

In addition, 3.5 μl of LRRK2^RCKW^:E11 complex was applied to glow-discharged UltrAuFoil 300 mesh R1.2/1.3 grids (Quantifoil, Q350AR13A) and incubated in a FEI Vitrobot IV at 4 °C and 95% humidity for 20 s. The grids were blotted for 4 s at an offset of −20 mm on both sides and vitrified by plunging into liquid ethane cooled down to liquid nitrogen temperature. Data were collected on an FEI Talos Arctica operated at 200 kV and equipped with a K2 summit direct electron detector (Gatan), using Leginon (version 1.0, http://emg.nysbc.org/redmine/projects/leginon/wiki/Leginon_Homepage, RRID:SCR_015749) (39). Images were acquired at defocus values varying between −1.0 and −2.0 μm at a nominal magnification of 36000×, yielding a pixel size of 1.16 Å. The camera was operated in dose-fractionation counting mode collecting 40 frames per movie, with a total dose of 60 e^-^ per Å^2^. Our current protocol can be found at (dx.doi.org/10.17504/protocols.io.bp2l6224kgqe/v1).

### Cryo-EM data processing

In total, 2468 movies were collected and aligned using MotionCor2 (version 1.4, https://emcore.ucsf.edu/cryoem-software, RRID:SCR_016499) (40) and contrast transfer function parameters were estimated using Ctffind4 (version 4.0, http://grigoriefflab.janelia.org/ctffind4, RRID:SCR_016732) (41). Micrographs with a contrast transfer function estimation worse than 5 Å were discarded. Particles obtained with blob picker in CryoSparc (version 4.3, https://cryosparc.com/, RRID:SCR_016501) (42) were used to train a Topaz model version 0.2.5, https://cb.csail.mit.edu/cb/topaz/) (43). After inspection, particle extraction and several rounds of 2D classification, approximately 280,000 particles were extracted with a 288-pixel box and used for subsequent processing. After *ab initio* and heterogeneous refinement to separate particles with and without DARPin E11, 185,601 particles were selected and used as an input for a Non-Uniform Refinement. A final local refinement using a mask for the C-lobe of the kinase, the WD40 domain and E11 DARPin yielded a map at 3.6 Å.

### Model building

The available structure of LRRK2^RCKW^ (Protein Data Bank (PDB) ID 6VP7) was split into domains and fitted into the 3D maps using UCSF ChimeraX (version 1.5, https://www.cgl.ucsf.edu/chimerax/, RRID:SCR_015872) (44). For DARPin E11, we generated an initial model using ColabFold (45) (https://github.com/sokrypton/ColabFold) and used it as starting point for model building, which was performed in Coot (version 0.8.9, http://www2.mrc-lmb.cam.ac.uk/personal/pemsley/coot/, RRID:SCR_014222) (46). The built structures were refined using real-space refinement in Phenix (version 1.20, https://www.phenix-online.org/, RRID:SCR_014224) (47). Refinement statistics are summarized in Table S1. Figures were prepared using USCF ChimeraX (44).

### Kinase activity assay

To investigate the impact of DARPin E11 binding on LRRK2 kinase activity, a MS-based activity assay was performed. Phosphorylation of the physiological LRRK2 substrate Rab8A was measured. The reaction was set up as follows: 5 μM recombinant Rab8A was incubated with 50 nM LRRK2^RCKW^ and a concentration series of the respective DARPin ranging from 0.016 to 1 μM in reaction buffer (20 mM Hepes pH 7.4, 100 mM NaCl, 0.5 mM tris(2-carboxyethyl)phosphine (TCEP), 0.5% glycerol, 20 μM GDP, and 2.5 mM MgCl_2_). The phosphorylation reaction was started by adding ATP to the final 1 mM concentration. After incubation at RT for 3.5 h the reaction was stopped by adding an equal volume of MS buffer (dH_2_O with 0.1% formic acid). The Rab8A phosphorylation grade was analyzed by MS with an Agilent 6230 Electrospray Ionization Time-of-Flight MS coupled with the liquid chromatography unit 1260 Infinity. The sample was applied *via* a C3 column and eluted at 0.4 ml/min flow rate using a solvent gradient of water to acetonitrile with 0.1% formic acid. Data were acquired with the MassHunter LC/MS Data Acquisition software and analyzed with the BioConfirm vB.08.00 tool (both Agilent Technology, https://www.agilent.com/en/product/software-informatics/mass-spectrometry-software, RRID: SCR_016657). The relative kinase activity was plotted against the inhibitor concentration and fitted to a three parameter logistic model according to the least square method (GraphPad Prism 8.0). Our current protocol for this assay can also be found at (dx.doi.org/10.17504/protocols.io.6qpvr385ovmk/v2).

### Immunofluorescence, confocal microscopy, and image analysis

293T cells were plated on fibronectin-coated glass 22 mm × 22 mm coverslips and grown for 24 h before transfection with polyethylenimine (PEI) and 800 ng of GFP-LRRK2 alone or cotransfected with 400 ng 8xHis-DARPinE11-3xFLAG (in pCDNA3.1). After 24 to 48 h, the cells were incubated at 37  °C with DMSO or MLi-2 (Tocris, catalog number 5756; 2 μM) for 2 h. The cells were fixed with prewarmed 3% paraformaldehyde, 4% sucrose, in 1× PBS for 10 minutes at RT. The coverslips were then rinsed twice and washed twice with 1× PBS and quenched with 0.4% NH_4_Cl for 10 min. After washing with PBS, the cells were incubated with blocking and permeabilizing buffer (2% BSA, 0.1% Triton X-100 in 1× PBS) for 20 min at RT. A primary Rabbit anti-FLAG polyclonal antibody (ptg labs, 20543-1-AP, Lot # 00106090) was diluted 1:200 in antibody dilution buffer (2% BSA in 1× PBS) and incubated with the cells for 3 h at RT. Following primary antibody incubation, the coverslips were washed three times with 2% BSA in PBS 1× and incubated with 1:500 goat anti-rabbit Alexa568 secondary antibody (Invitrogen, A-11011, Lot # 568 2192277, diluted in antibody dilution buffer) for 1 h at RT. After secondary antibody incubation, the cells were treated with 1:1000 4′,6-diamidino-2-phenylindole (DAPI) in PBS for 10 min and then washed five times with 1× PBS before mounting on glass slides using FluorSave (EMD Millipore Sigma). The coverslips were left to dry at least 1 h and imaged immediately or stored at 4 °C for later imaging. Mounted cover slips were blinded before imaging to prevent bias in image acquisition. Imaging of blinded samples was done using a Yokogawa W1 confocal scan head mounted to a Nikon Ti2 microscope with an Apo ×60 1.49-NA objective. The microscope was run with NIS Elements using the 488 nm, 561 nm, and 405 nm lines of a six-line (405 nm, 445 nm, 488 nm, 515 nm, 561 nm, and 640 nm) LUN-F-XL laser engine and a Prime95B camera (Photometrics). A total of 16 to 20 areas were imaged per sample. Cells expressing both LRRK2 (positive for GFP fluorescence signal) and DARPin E11 (stained with anti- FLAG antibody) were assessed for the presence or absence of LRRK2 filaments in ImageJ2 (https://imagej.net/, version 2.14.0, RRID:SCR_003070). Maximum projections were generated from confocal z-stacks as a guide. The number of cells with filaments was divided by the total number of cells coexpressing LRRK2 and DARPin E11 to determine the percent of cells with filaments per replicate. Three or four replicates were obtained for each condition on at least two separate days. The slides were imaged blinded and analyzed by two different researchers. Unblinding took place only after all analyses were completed. Data were plotted, checked for normality with a D’Agostino-Pearson test and statistics were generated in GraphPad Prism (version 10.1.3, RRID:SCR_002798). Our current protocol for this assay can also be found at (dx.doi.org/10.17504/protocols.io.8epv5xpz4g1b/v1).

### LRRK2 kinase assay in cells

293T cells (American Type Culture Collection, ATCC cat. no. CRL-3216, RRID: CVCL_0063) were transfected with 1000 ng of WT, full-length, untagged LRRK2 (pCDNA5-LRRK2) and 500 ng GFP-Rab8A (Addgene; RRID: Addgene_49543) or 1000 ng LRRK2, 500 ng GFP-Rab8A, and 500 ng of 8xHis-DARPin E11-3xFLAG using polyethylenimine. After 48 h, cells transfected only with LRRK2 and GFP-Rab8A (without DARPin E11) were incubated at 37 °C, 5% CO2 with either DMSO or MLi-2 (Tocris, catalog number 5756; 2 μM) for 1 h. Cells were washed twice with RT PBS one time and a final time with cold PBS. Cells were lysed in cold RIPA lysis buffer (50 mM Tris pH 8.0, 150 mM NaCl, 1% Triton X-100, 0.1% SDS, 0.5% sodium deoxycholate, and 1 mM DTT) in the presence of cOmplete mini EASYpack protease inhibitor cocktail and the PhosSTOP EASYpack phosphatase inhibitor cocktail (Roche). Resuspended cells were vortexed for 30 s six times and incubated on a rotator for 15 min at 4 °C. Cell lysates were then spun down at 13,000*g* for 15 min at 4 °C. SDS PAGE loading buffer was added to the supernatant and was either used immediately or stored at −20 °C. Afterward, 15 μl of sample were loaded into a NuPAGE 4 to 12% gradient Bis-Tris gel (Invitrogen). Protein was transferred onto polyvinylidene fluoride (PVDF) transfer membrane (0.45 μm pore size) for 100 min at 200 mA at 4 °C. The membrane was cut directly above the 70 kDa molecular weight marker band to separate LRRK2 from the rest of the membrane. The upper portion of the membrane containing LRRK2 was blocked with 5% BSA for 1 h, at RT and incubated with 1:1000 rabbit anti-LRRK2 monoclonal antibody (MJFF2 (c41–2); Abcam ab133474, Lot # GR3445288–3; RRID:AB_2713963) diluted in 1% BSA overnight at 4 °C. The lower part of the membrane (below 70 kDa) was blocked in 5% milk for 1 h at RT and incubated with a monoclonal mouse anti-GFP antibody (1:2500, Santa Cruz Biotechnology sc-9996, Lot # C2523; RRID:AB_627695), a rabbit anti-pRab8A phospho T72 monoclonal antibody (1:1000, Abcam 230260, Lot #GR3216587–1;RRID:AB_2814988), and a rabbit anti-GAPDH monoclonal antibody (Cell Signaling Technology 2118, Lot # 16, 1:3000; RRID:AB_561053), diluted in 1% milk, overnight at 4 °C. The next day, the membranes were washed once with Tris buffered saline buffer with 0.05% Tween-20 (TBS-T) for 10 min and three times at RT with TBS-T before the secondary antibodies were added. A secondary goat anti-rabbit Licor680 antibody (LI-COR biosciences; catalog number 926-32211, Lot # D40109-05; RRID:AB_621843) was diluted 1:5000 in 4% BSA and added to the top LRRK2-blotted membrane and incubated at RT for 1 h. Secondary goat anti-rabbit Licor680 and goat anti-mouse Licor800 (LiCOR Biosciences, catalog number 926-68070 Lot # D30418-25; RRID: AB_10956588) were diluted 1:5000 in 4% milk and incubated with the lower half of the membrane for 1 h at RT. Membranes were washed for 10 min two times with TBS-T and once with TBS, and then imaged on an Odyssey CLx Li-Cor imaging system. After imaging, the lower half of the membrane was blocked again for 1 h at RT with 5% milk and then incubated with primary rabbit anti-FLAG polyclonal antibody (ptg labs 20543-1-AP, Lot # 00106090; RRID:AB_11232216) diluted 1:500 in 1% milk overnight at 4 °C. The membrane was washed as before, then incubated at RT with secondary goat anti-rabbit Licor680 diluted 1:5000 in 4% milk. After washing again in TBS-T, the membrane was reimaged on the Li-Cor imaging system. Images were processed with ImageJ (version 2.14.0). The difference in phosphorylation of Rab8A between DMSO, MLi-2, and DARPin E11 conditions was analyzed using an one-way ANOVA and corrected using Tukey’s multiple comparison test. All statistical analyses were performed using GraphPad Prism (version 10.1.3, RRID: SCR_002798). Our current protocol can also be found at dx.doi.org/10.17504/protocols.io.kxygx9bozg8j/v1.

## Data availability

The cryo-EM map and model for LRRK2^RCKW^:E11 have been deposited in the EM and Protein Data Banks, respectively. Accession codes are 8U1B (PDB) and EMBD-41806 (EMDB).

## Supporting information

This article contains supporting information.

## Conflicts of interest

SRP is a consultant for Schrodinger and Stoke Therapeutics. AP is a co-founder and shareholder of Molecular Partners who are commercializing the DARPin technology. All other authors do not have any conflicts to report with the contents of this article.

## References

[1] Paisán-Ruíz C., Jain S., Evans E.W., Gilks W.P., Simón J., van der Brug M. (2004). Cloning of the gene containing mutations that cause PARK8-linked Parkinson’s disease. Neuron.

[2] Zimprich A., Biskup S., Leitner P., Lichtner P., Farrer M., Lincoln S. (2004). Mutations in LRRK2 cause autosomal-dominant parkinsonism with pleomorphic pathology. Neuron.

[3] Deniston C.K., Salogiannis J., Mathea S., Snead D.M., Lahiri I., Matyszewski M. (2020). Structure of LRRK2 in Parkinson’s disease and model for microtubule interaction. Nature.

[4] Myasnikov A., Zhu H., Hixson P., Xie B., Yu K., Pitre A. (2021). Structural analysis of the full-length human LRRK2. Cell.

[5] Zhang P., Fan Y., Ru H., Wang L., Magupalli V.G., Taylor S.S. (2019). Crystal structure of the WD40 domain dimer of LRRK2. Proc. Natl. Acad. Sci. U. S. A..

[6] Blanca Ramírez M., Lara Ordóñez A.J., Fdez E., Madero-Pérez J., Gonnelli A., Drouyer M. (2017). GTP binding regulates cellular localization of Parkinson’s disease-associated LRRK2. Hum. Mol. Genet..

[7] Kett L.R., Boassa D., Ho C.C.-Y., Rideout H.J., Hu J., Terada M. (2012). LRRK2 Parkinson disease mutations enhance its microtubule association. Hum. Mol. Genet..

[8] Watanabe R., Buschauer R., Böhning J., Audagnotto M., Lasker K., Lu T.-W. (2020). The in situ structure of Parkinson’s disease-linked LRRK2. Cell.

[9] Gloeckner C.J., Kinkl N., Schumacher A., Braun R.J., O’Neill E., Meitinger T. (2005). The Parkinson Disease causing LRRK2 mutation I2020T is associated with increased kinase activity. Hum. Mol. Genet..

[10] Sheng Z., Zhang S., Bustos D., Kleinheinz T., Le Pichon C.E., Dominguez S.L. (2012). Ser1292 autophosphorylation is an indicator of LRRK2 kinase activity and contributes to the cellular effects of PD mutations. Sci. Transl. Med..

[11] Steger M., Tonelli F., Ito G., Davies P., Trost M., Vetter M. (2016). Phosphoproteomics reveals that Parkinson’s disease kinase LRRK2 regulates a subset of Rab GTPases. eLife.

[12] West A.B., Moore D.J., Biskup S., Bugayenko A., Smith W.W., Ross C.A. (2005). Parkinson’s disease-associated mutations in leucine-rich repeat kinase 2 augment kinase activity. Proc. Natl. Acad. Sci. U. S. A..

[13] Taymans J.-M., Fell M., Greenamyre T., Hirst W.D., Mamais A., Padmanabhan S. (2023). Perspective on the current state of the LRRK2 field. NPJ Parkinsons. Dis..

[14] Fell M.J., Mirescu C., Basu K., Cheewatrakoolpong B., DeMong D.E., Ellis J.M. (2015). MLi-2, a potent, selective, and centrally active compound for exploring the therapeutic potential and safety of LRRK2 kinase inhibition. J. Pharmacol. Exp. Ther..

[15] Estrada A.A., Chan B.K., Baker-Glenn C., Beresford A., Burdick D.J., Chambers M. (2014). Discovery of highly potent, selective, and brain-penetrant aminopyrazole leucine-rich repeat kinase 2 (LRRK2) small molecule inhibitors. J. Med. Chem..

[16] Schmidt S.H., Weng J.-H., Aoto P.C., Boassa D., Mathea S., Silletti S. (2021). Conformation and dynamics of the kinase domain drive subcellular location and activation of LRRK2. Proc. Natl. Acad. Sci. U. S. A..

[17] Liu X., Kalogeropulou A.F., Domingos S., Makukhin N., Nirujogi R.S., Singh F. (2022). Discovery of XL01126: a potent, fast, cooperative, selective, orally bioavailable, and blood-brain barrier penetrant PROTAC degrader of leucine-rich repeat kinase 2. J. Am. Chem. Soc..

[18] Hatcher J.M., Zwirek M., Sarhan A.R., Vatsan P.S., Tonelli F., Alessi D.R. (2023). Development of a highly potent and selective degrader of LRRK2. Bioorg. Med. Chem. Lett..

[19] Li T., He X., Thomas J.M., Yang D., Zhong S., Xue F. (2015). A novel GTP-binding inhibitor, FX2149, attenuates LRRK2 toxicity in Parkinson’s disease models. PLoS One.

[20] Chaikuad A., Keates T., Vincke C., Kaufholz M., Zenn M., Zimmermann B. (2014). Structure of cyclin G-associated kinase (GAK) trapped in different conformations using nanobodies. Biochem. J..

[21] Singh R.K., Soliman A., Guaitoli G., Störmer E., von Zweydorf F., Dal Maso T. (2022). Nanobodies as allosteric modulators of Parkinson’s disease-associated LRRK2. Proc. Natl. Acad. Sci. U. S. A..

[22] Plückthun A. (2015). Designed ankyrin repeat proteins (DARPins): binding proteins for research, diagnostics, and therapy. Annu. Rev. Pharmacol. Toxicol..

[23] Schilling J., Schöppe J., Plückthun A. (2014). From DARPins to LoopDARPins: novel LoopDARPin design allows the selection of low picomolar binders in a single round of ribosome display. J. Mol. Biol..

[24] Dreier B., Plückthun A. (2012). Rapid selection of high-affinity binders using ribosome display. Methods Mol. Biol..

[25] Plückthun A. (2012). Ribosome display: a perspective. Methods Mol. Biol..

[26] Kummer L., Hsu C.-W., Dagliyan O., MacNevin C., Kaufholz M., Zimmermann B. (2013). Knowledge-based design of a biosensor to quantify localized ERK activation in living cells. Chem. Biol..

[27] Strubel A., Münick P., Chaikuad A., Dreier B., Schaefer J., Gebel J. (2022). Designed Ankyrin Repeat Proteins as a tool box for analyzing p63. Cell Death Differ..

[28] Qi C., Lavriha P., Mehta V., Khanppnavar B., Mohammed I., Li Y. (2022). Structural basis of adenylyl cyclase 9 activation. Nat. Commun..

[29] Liu Y., Gonen S., Gonen T., Yeates T.O. (2018). Near-atomic cryo-EM imaging of a small protein displayed on a designed scaffolding system. Proc. Natl. Acad. Sci. U. S. A..

[30] Vulovic I., Yao Q., Park Y.-J., Courbet A., Norris A., Busch F. (2021). Generation of ordered protein assemblies using rigid three-body fusion. Proc. Natl. Acad. Sci. U. S. A..

[31] Yao Q., Weaver S.J., Mock J.-Y., Jensen G.J. (2019). Fusion of DARPin to aldolase enables visualization of small protein by cryo-EM. Structure.

[32] Blanc M., Lettl C., Guérin J., Vieille A., Furler S., Briand-Schumacher S. (2023). Designed Ankyrin Repeat Proteins provide insights into the structure and function of CagI and are potent inhibitors of CagA translocation by the Helicobacter pylori type IV secretion system. PLoS Pathog..

[33] Gilbreth R.N., Koide S. (2012). Structural insights for engineering binding proteins based on non-antibody scaffolds. Curr. Opin. Struct. Biol..

[34] Sanz Murillo M., Villagran Suarez A., Dederer V., Chatterjee D., Alegrio Louro J., Knapp S., Mathea S., Leschziner A.E. (2023). Inhibition of Parkinson’s disease–related LRRK2 by type I and type II kinase inhibitors: activity and structures. Sci. Adv..

[35] Steger M., Diez F., Dhekne H.S., Lis P., Nirujogi R.S., Karayel O. (2017). Systematic proteomic analysis of LRRK2-mediated Rab GTPase phosphorylation establishes a connection to ciliogenesis. eLife.

[36] Snead D.M., Matyszewski M., Dickey A.M., Lin Y.X., Leschziner A.E., Reck-Peterson S.L. (2022). Structural basis for Parkinson’s disease-linked LRRK2’s binding to microtubules. Nat. Struct. Mol. Biol..

[37] Clarimón J., Pagonabarraga J., Paisán-Ruíz C., Campolongo A., Pascual-Sedano B., Martí-Massó J.-F. (2008). Tremor dominant parkinsonism: clinical description and LRRK2 mutation screening. Mov. Disord..

[38] Kalogeropulou A.F., Purlyte E., Tonelli F., Lange S.M., Wightman M., Prescott A.R. (2022). Impact of 100 LRRK2 variants linked to Parkinson’s disease on kinase activity and microtubule binding. Biochem. J..

[39] Suloway C., Pulokas J., Fellmann D., Cheng A., Guerra F., Quispe J. (2005). Automated molecular microscopy: the new Leginon system. J. Struct. Biol..

[40] Zheng S.Q., Palovcak E., Armache J.-P., Verba K.A., Cheng Y., Agard D.A. (2017). MotionCor2: anisotropic correction of beam-induced motion for improved cryo-electron microscopy. Nat. Methods.

[41] Rohou A., Grigorieff N. (2015). CTFFIND4: fast and accurate defocus estimation from electron micrographs. J. Struct. Biol..

[42] Punjani A., Rubinstein J.L., Fleet D.J., Brubaker M.A. (2017). cryoSPARC: algorithms for rapid unsupervised cryo-EM structure determination. Nat. Methods.

[43] Bepler T., Morin A., Rapp M., Brasch J., Shapiro L., Noble A.J. (2019). Positive-unlabeled convolutional neural networks for particle picking in cryo-electron micrographs. Nat. Methods..

[44] Pettersen E.F., Goddard T.D., Huang C.C., Meng E.C., Couch G.S., Croll T.I. (2021). UCSF ChimeraX: structure visualization for researchers, educators, and developers. Protein Sci..

[45] Mirdita M., Schütze K., Moriwaki Y., Heo L., Ovchinnikov S., Steinegger M. (2022). ColabFold: making protein folding accessible to all. Nat. Methods.

[46] Emsley P., Lohkamp B., Scott W.G., Cowtan K. (2010). Features and development of coot. Acta Crystallogr. D Biol. Crystallogr..

[47] Liebschner D., Afonine P.V., Baker M.L., Bunkóczi G., Chen V.B., Croll T.I. (2019). Macromolecular structure determination using X-rays, neutrons and electrons: recent developments in Phenix. Acta Crystallogr. D Struct. Biol..

