# Supplementary material for: A designed ankyrin-repeat protein that targets Parkinson’s disease-associated LRRK2
Source: J Biol Chem. 2024 Jun 12;300(7):107469. doi: 10.1016/j.jbc.2024.107469 (PMC11284679; doi:10.1016/j.jbc.2024.107469)
Supplement: Supporting information [file mmc1.pdf]

Supporting Information

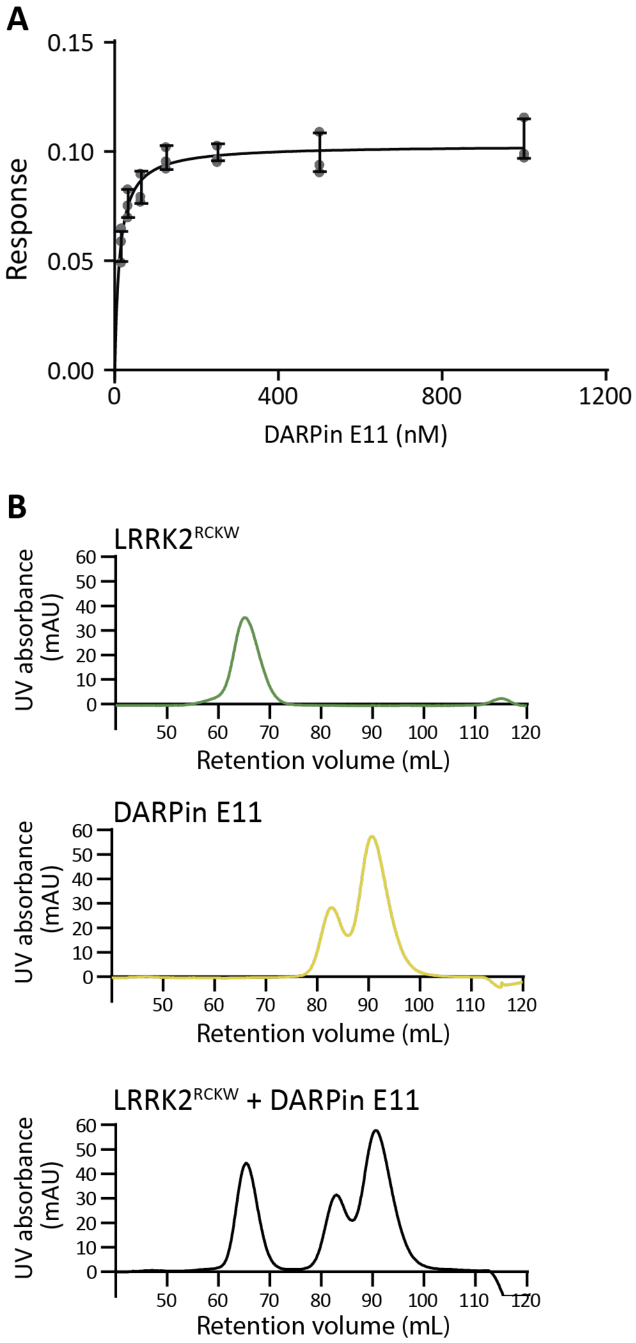

Figure S1. DARPin E11 binds to LRRK2^{RCKW} with high affinity.

A. Bio-layer interferometry analysis of the LRRK2^{RCKW}:E11 interaction yielded a K_D value of 11 nM ± 2 nM (SD). The response was plotted against the E11 concentration and fitted to the Langmuir equation according to the least square method. Plot points represent data from three technical replicates. **B.** The LRRK2^{RCKW}:E11 complex was subjected to size exclusion chromatography (SEC). Chromatographs are shown, which correspond to the SDS PAGE gels shown in Figure 1.

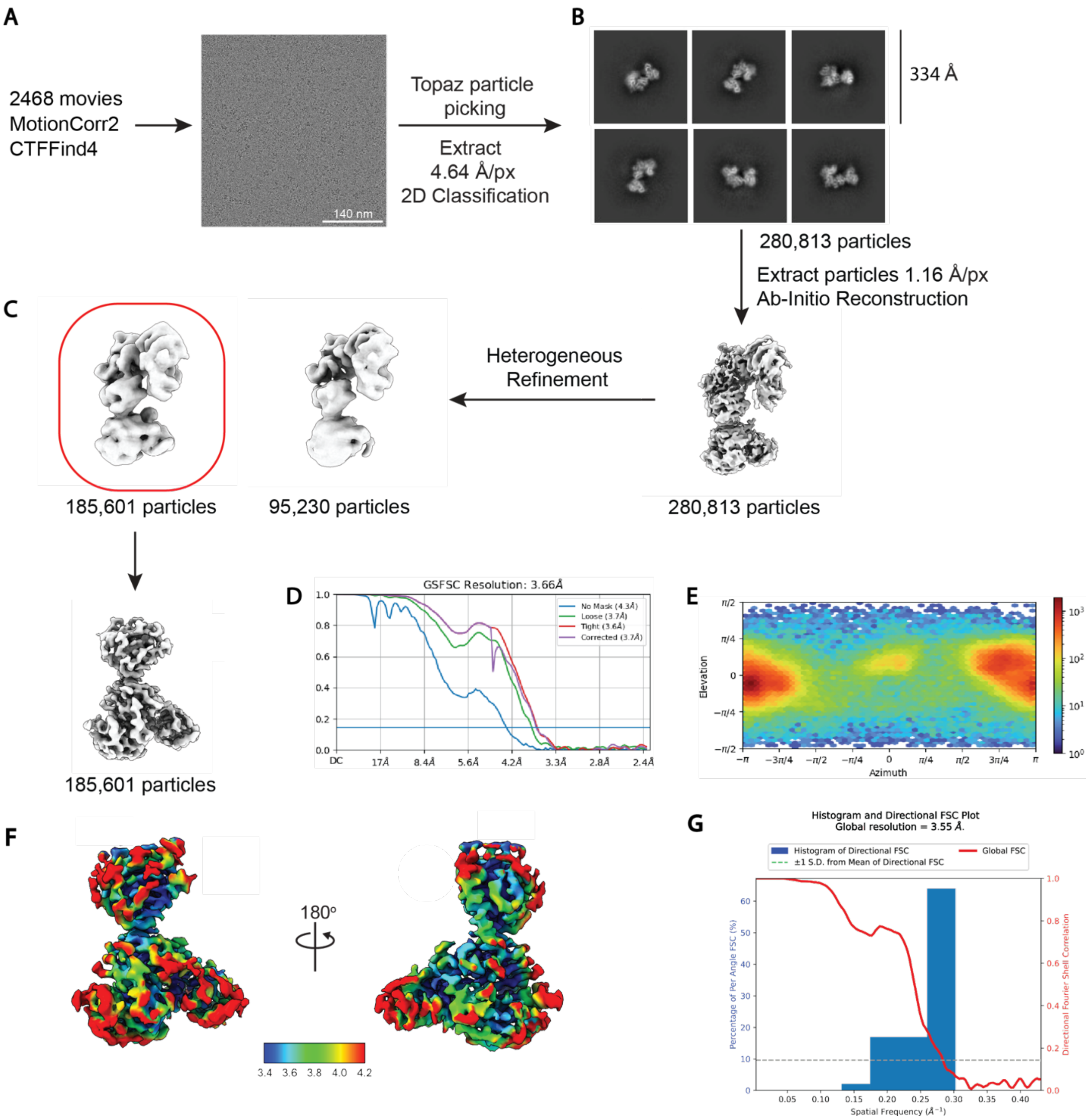

Figure S2. Cryo-EM data processing workflow for LRRK2^{RCKW}:E11 DARPin.
A. An initial dataset with 2,468 movies was collected from a sample of the LRRK2^{RCKW}:E11 DARPin complex. A typical micrograph is shown. Scale bar, 140 nm. **B.** 2D class averages. Scale bar, 334 Å. **C.** Data processing strategy. All processing was done in CryoSPARC. **D.** FSC curves. **E.** Euler angle distribution. **F.** Local Resolution map. **G.** 3D-FSC analysis including global half map FSC (red line) and histogram of values evenly sampled over the 3D FSC (blue bars).

Table S1. Cryo-EM data collection, refinement and validation statistics.

	$LRRK2^{KW}$:DARPin E11 (EMDB-41806, PDB 8U1B)
**Data collection and processing**	
Magnification	36000
Voltage (kV)	200
Electron exposure (e–/Å^2)	52
Defocus range (μm)	-1.0 to -2.5
Pixel size (Å)	1.16
Symmetry imposed	C1
Initial particle images (no.)	280,813
Final particle images (no.)	185,601
Map resolution (Å)	3.66
FSC threshold	0.143
Map resolution range (Å)	3.4 – 4.2
**Refinement**	
Initial model used (PDB code)	6VP7
Model resolution (Å)	3.66
FSC threshold	0.143
Model composition	
Non-hydrogen atoms	4540
Protein residues	604
B factors (Å^2)	
Protein	778.59
R.m.s. deviations	
Bond lengths (Å)	0.009
Bond angles (°)	1.338
Validation	
MolProbity score	2.34
Clashscore	18.15
Ramachandran plot	
Favored (%)	91.29
Allowed (%)	8.36
Disallowed (%)	0.35
